# Human recombinant anti-thyroperoxidase autoantibodies: *in vitro* cytotoxic activity on papillary thyroid cancer expressing TPO

**DOI:** 10.1038/sj.bjc.6605464

**Published:** 2010-02-09

**Authors:** S A Rebuffat, M Morin, B Nguyen, F Castex, B Robert, S Péraldi-Roux

**Affiliations:** 1 CNRS-UMR 5232, CPID, Faculté de Pharmacie, 15 avenue Charles Flahault, BP 14491, 34093 Montpellier Cedex 5, France; 2 INSERM-U896, IRCM Val d'Aurelle, UM1, 34298 Montpellier, France

**Keywords:** thyroid cancer, anti-TPO antibodies, cytotoxicity, immunotherapy

## Abstract

**Background:**

Thyroid cancers are difficult to treat due to their limited responsiveness to chemo- and radiotherapy. There is thus a great interest in and a need for alternative therapeutic approaches.

**Results:**

We studied the cytotoxic activity of anti-thyroperoxidase autoantibodies (anti-TPO aAbs, expressed in baculovirus/insect cell (B4) and CHO cells (B4′) or purified from patients' sera) against a papillary thyroid cancer (NPA) cell line. Anti-TPO aAbs from patients' sera led to a partial destruction of NPA cell line by complement-dependent cytotoxicity (CDC) and antibody-dependent cell-mediated cytotoxicity (ADCC) and exhibited an anti-proliferative activity. Comparison of the cytotoxic activity of anti-TPO aAbs shows that B4′ induced an anti-proliferative effect and a better ADCC than B4, but a lower one than anti-TPO aAbs from patients' sera. Antibody-dependent cell-mediated cytotoxicity was increased when human peripheral blood mononuclear cells were used as effector cells, suggesting that Fc*γ*Rs, CD64, CD32 and CD16 are involved. Indeed, anti-TPO aAbs from patients' sera, but not B4 and B4′, exhibited CDC activity.

**Conclusions:**

These data indicate that anti-TPO aAbs display moderate ADCC and anti-proliferative activities on NPA cells; IgG glycosylation appears to be important for cytotoxic activity and ADCC efficiency depends on Fc*γ*R-bearing cells. Finally, recombinant human anti-TPO aAbs cannot yet be considered as an optimal tool for the development of a novel therapeutic approach for thyroid cancer.

Thyroid carcinoma accounts for roughly 1% of all new malignant tumours, and a majority of thyroid cancers are well differentiated, including papillary, follicular and Hürthle cell carcinoma. In most patients, the management of well-differentiated thyroid cancer is partial or complete thyroidectomy in conjunction with chemo- or radioiodine therapy. However, a substantial number of patients with differentiated thyroid cancer do not respond to radioiodine therapy, and 5–20% of them develop lung metastases, which, in most cases, do not respond to radioiodine therapy and are inoperable. Therefore, new complementary therapeutic approaches could be of interest in the treatment of thyroid carcinomas.

Thyroperoxidase (TPO) is expressed on the apical membrane of both normal and malignant thyroid cells. Indeed, unlike other thyroid-specific antigens, such as thyroglobulin and TSH receptor that are no longer expressed in thyroid carcinomas, TPO expression persists even on poorly differentiated thyroid tumours (Czarnocka *et al*, 1985). Therefore, TPO expressed at the surface of thyroid cancer cells can be recognised by anti-TPO autoantibodies (aAbs) and might constitute a potential target for antibody-specific immunotherapy. Thyroperoxidase is one of the major autoantigens involved in autoimmune thyroid diseases (AITD), such as Hashimoto's thyroiditis (McLachlan and Rapoport, 1992, 2000). Hashimoto's thyroiditis is characterised by glandular hypo-function, destruction of thyroid cells and production of an immunoglobulin G (IgG) response directed against TPO. Anti-TPO aAbs are invaluable markers of thyroid autoimmune response and have been shown to exert both *in vitro* and *in vivo* cytotoxic functions such as C3 complement activation (Wadeleux *et al*, 1989; Chiovato *et al*, 1993; Parkes *et al*, 1994) and antibody-dependent cell-mediated cytotoxicity (ADCC) (Bogner *et al*, 1989; Rodien *et al*, 1996; Guo *et al*, 1997; Metcalfe *et al*, 1997; Rebuffat *et al*, 2008). These effects probably participate in the maintenance and amplification of thyroid cell destruction in Hashimoto's disease. On the basis of these observations, one could envision the use of anti-TPO aAbs as potential antigen-specific agents with cytotoxic activities for the treatment of thyroid carcinomas.

In addition, it is well known that in AITD such as Hashimoto's disease, lymphocytes infiltrate and destroy thyroid gland. Previous reports have suggested that the immune system attempts to control cancer thyroid cells. Indeed, lymphocytes as well as monocytes/macrophages are often found in the periphery or infiltrated in the tumours, which make them potential effectors involved in the destruction of cancer cells.

Using the phage-display technology, we previously selected and characterised human recombinant anti-TPO aAbs (Chapal *et al*, 2000, 2001). These human recombinant anti-TPO aAbs mimic human anti-TPO aAbs present in the sera of patients suffering from AITD and recognise the human TPO (hTPO) immunodominant region (Bresson *et al*, 2003, 2004, 2005, Rebuffat *et al*, 2006). To evaluate if these anti-TPO aAbs may be useful tools in the treatment of thyroid carcinomas, we selected a human recombinant anti-TPO aAb and expressed it in baculovirus (B4) and in CHO cells (B4′). We explored their biological properties *in vitro* before future *in vivo* preclinical tests. Cytotoxic activity, complement-dependent cytotoxicity (CDC) and ADCC, of purified human recombinant anti-TPO aAbs (B4 and B4′) expressed in respectively baculovirus and CHO cells, were studied on thyroid carcinoma cells and compared with those of circulating anti-TPO aAbs purified from the sera of patients suffering from AITD, using the same target cells. We show here that anti-TPO aAb B4′ purified from CHO is able to induce a moderate cytotoxic activity, lower than that of patients' circulating anti-TPO aAbs on the papillary carcinoma cell line NPA, whatever the effector cells used (peripheral blood mononuclear cells (PBMC) or monocyte cell line). However, neither human recombinant anti-TPO aAbs B4 purified from baculovirus/insect cells nor deglycosylated aAbs from patients' sera appear able to induce any significant CDC, ADCC or anti-proliferative activity.

## Materials and methods

### Reagents

Human recombinant anti-TPO scFv antibody B_4_ was selected in our laboratory using a phage-display library and expressed as IgG_1_ in baculovirus/insect cells system by Dr M Cerutti as previously described (Bresson *et al*, 2001, 2003). Sera from patients suffering from Graves' disease were obtained from Dr B Guerrier (Guy de Chauliac Hospital, Montpellier, France) and Dr L Baldet (Lapeyronie University Hospital, Montpellier, France). IgG_1_ antibodies were purified on a protein G affinity column and the concentration determined by absorbance at 280 nm, E^0.1%^ of 1.40. Human sera anti-TPO aAbs were deglycosylated using the Dig Glycan differentiation kit (Roche, Basel, Switzerland). Mouse monoclonal anti-hTPO antibodies mAb15 and mAb47 were provided by Dr J Ruf (INSERM Unit 555, Marseille, France). Human TPO, purified (more than 95% pure) from thyroid glands, was obtained from HyTest Ltd (Turku, Finland).

### Cells

Human anaplastic thyroid cancer cell lines SW1736 and C643 (Mark *et al*, 1987) were kindly provided by Dr NE Heldin, Denmark, and human follicular thyroid cancer cell lines ML1 (Schonberger *et al*, 2000) and WRO (Estour *et al*, 1989) were from J Schonberger. The human papillary thyroid cancer cell line NPA was a jpegt from Professor Santoro, Italy (Fagin *et al*, 1993). Cells were cultured in RMPI 1640 (Cambrex, Belgium) containing 10 % fetal calf serum (FCS) (PAA Laboratories, Linz, Austria), 2 *μ*M L-glutamine, 100 U ml^−1^ of penicillin and 100 *μ*g ml^−1^ of streptomycin (Life Technologies Inc., Paisley, UK).

Peripheral blood mononuclear cells obtained from healthy donors (Etablissement Français du Sang, Montpellier, France) were separated from blood by Ficoll density gradient (Histopaque-1077; Sigma-Aldrich, St Louis, MO, USA) and resuspended in RPMI 1640 supplemented with 10% heat-inactivated FCS, 50 U ml^−1^ penicillin, 50 *μ*g ml^−1^ streptomycin and 2 mmol l^−1^ glutamine.

The 11A1.6 CD64^+^ (van Vugt *et al*, 1999), CDw32-L CD32^+^ (Peltz *et al*, 1988) and Jurkat CD16^+^ (Vivier *et al*, 1992) cell lines were kindly provided by Professor J van de Winkel, Dr J Lewsen (Immunotherapy Laboratory, Utrech University, The Netherlands), Dr JP Vendrell (Virology Laboratory, University Hospital, Montpellier, France) and Professor E Vivier (INSERM U608, Marseille, France) respectively.

The effector cell line (HL-60), a human monocyte line (provided by J Dornand, Montpellier), was grown in the same medium as PBMC. Five days before the experiment, 1,25-dihydroxyvitamin D_3_ (1,25(OH)_2_D_3_) (=D_3_ vitamin) was added to induce differentiation of HL-60 cells into monocytes. All cell cultures were incubated at 37°C in a humidified atmosphere containing 5% CO_2_. Methotrexate (5 *μ*M; Sigma-Aldrich) and geneticin (G418) (0.5 mg ml^−1^; Sigma-Aldrich) were added, respectively, to 11A.6 CD64^+^ and Jurkat CD16^+^ cell lines.

The expression of CD14, CD16 (Fc*γ*RIII), CD32 (Fc*γ*RII), CD64 (Fc*γ*RI) on the surface of the different effector cells (HL-60 and PBMC) was investigated using fluorescein-conjugated anti-human CD Ab (Miltenyi Biotech, Bergisch Gladbach, Germany and Pharmingen, Becton Dickinson, Franklin Lakes, NJ, USA) and analysed by flow cytometry analysis as previously described (Rebuffat *et al*, 2008). Monocytes and lymphocytes present in PBMC were identified by size/structure analysis.

### Expression and production of recombinant human anti-TPO antibody in CHO cells

The recombinant human anti-TPO scFv B4 was selected from an antibody phage-display library obtained from B cells extracted from thyroid tissue of patients suffering from Graves' disease (Chapal *et al*, 2001). ScFv B4 was expressed as a whole IgG_1_ antibody in the baculovirus/insect cell system (anti-TPO recombinant aAb B4). To improve glycosylation of the Fc part of this recombinant antibody, we also expressed scFv B4 as an IgG_1_ in the CHO eukaryote system (anti-TPO recombinant aAb B4′). The variable heavy chain of B4 was cloned between *Bam*HI and *Nhe*I restriction sites in the plasmid vector pcDNA3 (Invitrogen Life Technologies, Carlsbad, CA, USA) containing the sequence of the heavy chain (pcDNA3/H) and the variable light chain of B4 between *Bam*HI and *Xho*I in another plasmid vector pcDNA3 containing the sequence of the light chain (pcDNA3/L). These two plasmid vectors were kindly provided by Stephane Birkle (Alvarez-Rueda *et al*, 2007). Expression plasmids pcDNA3/L and pcDNA3/H were transfected into CHO cells using a Polyfect Transfection Reagent kit (Qiagen GmbH, Germany) according to the manufacturer's instructions. Cells were selected in the presence of 0.5 mg ml^−1^ G418 and 0.5 mg ml^−1^ Zeocin (Invitrogen Life Technologies). Double-resistant clones were isolated by limiting dilution, and the supernatants of resistant transfectants screened by ELISA. The anti-TPO recombinant aAb B4′ IgG_1_ was purified on a protein G affinity column and the concentration determined by measuring the absorbance at 280 nm, E^0.1%^ of 1.40.

### Flow cytometry analysis of TPO expression on the membrane of human thyroid cancer cell lines

Cells were removed from the culture flasks using HEPES–EDTA buffer (HEPES 10 mM, EDTA 3 mg ml^−1^, pH 7.0), rinsed and pelleted (5 min, 1000 r.p.m., 4°C) in Dulbecco's phosphate-buffered saline (D-PBS) (Cambrex) containing 2% FCS (=F-buffer). Approximately 10^6^ cells were incubated with 200 *μ*l of F-buffer containing 10 *μ*g ml^−1^ of human anti-TPO aAb for 60 min at 4°C, then washed twice and further incubated in 200 *μ*l of F-buffer containing 10 *μ*g ml^−1^ of fluorescein-conjugated anti-human IgG *γ*-chain-specific Ab (Sigma-Aldrich) for 60 min at 4°C in the dark. Negative controls were performed by incubating cells with only the secondary antibody. After two washings with D-PBS, the cells were analysed (10 000 events) with an EPICS XL4 (Beckman-Coulter, Fullerton, CA, USA) fluocytometer.

### Flow cytometry analysis of human anti-TPO antibodies bound to TPO expressed on the membrane of NPA cells

Flow cytometry analysis was carried out as previously described. NPA cells (∼10^6^) were incubated with human anti-TPO aAb (B4, B4′ or purified from sera of patients suffering from AITD); the reaction was revealed with a secondary antibody and analysed with an EPICS XL4 fluocytometer.

### Flow cytometric analysis of human anti-TPO antibodies bound to the Fc*γ*R

Cells expressing specifically one Fc*γ*R and effector cells (PBMC and HL-60) were rinsed and pelleted (5 min, 1000 r.p.m., 4°C) in F-buffer. The cells (∼10^6^) were incubated for 90 min at 4°C with 200 *μ*l of F-buffer containing 10 *μ*g ml^−1^ of the complex TPO/human anti-TPO aAb (B4, B4′ and aAbs purified from sera of patients suffering from AITD, preliminary incubated with hTPO (HyTest Ltd) for 45 min at 4°C). They were then washed, incubated with 10 *μ*g ml^−1^ of fluorescein-conjugated anti-human IgG *γ*-chain-specific antibody and binding analysed as previously described.

### *In vitro* antibody-dependent cell-mediated cytotoxicity assay

Antibody-dependent cell-mediated cytotoxicity assays were carried out using the standard ^51^Cr release assay (Rebuffat *et al*, 2008). Target cells (NPA cell line) were removed from culture flasks using HEPES–EDTA buffer, rinsed and aliquots (∼10^6^ cells) incubated (37°C, 60 min, 5% CO_2_) in culture medium 100 *μ*Ci ^51^Cr. Cells were then washed to eliminate unincorporated ^51^Cr and labelled target cells incubated (4°C, 45 min) with human anti-TPO antibodies (B4 and B4′ or aAbs purified from sera of patients suffering from AITD) (50 *μ*g ml^−1^). They were then seeded (2 × 10^4^ cells per well) in 96-well U-bottomed culture plates and incubated with effector cells (effector/target ratio, 50:1 PBMC, 12:1 HL-60). As controls, Jurkat cells were submitted to the same conditions than NPA cells and an irrelevant human IgG from healthy subjects or culture medium alone were used. Target cells were used to determine spontaneous ^51^Cr released in culture medium only and total ^51^Cr release was assessed by addition of HCl (1 N). Tests and controls were performed in triplicate. Cells were incubated for 6 h (37°C, 5% CO_2_), centrifuged (5 min, 1000 **g**) and aliquots (100 *μ*l) sampled for *γ*-counting. Cytotoxicity was expressed as specific ^51^Cr release calculated as follows: Lysis=((c.p.m. with effector cells) – (c.p.m. with culture medium))/((c.p.m. with HCl) – (c.p.m. with culture medium)).

### Complement fixation assay

To study the ability of TPO/anti-TPO antibody complexes to bind to C1q complement, microtitre plates were coated with TPO/anti-TPO aAbs (B4 and B4′ and aAbs purified from patients' sera) complexes, in 100 mM NaHCO_3_ (pH 9.0) at 4°C overnight. The plates were washed three times with D-PBS-Tween 0.1% and blocked with 1% non-fat powdered milk in D-PBS-Tween 0.1% (blocking buffer) for 60 min at 37°C. After washing, C1q complement was incubated in the blocking buffer for 90 min at 37°C. Plates were then washed and anti-C1q antibody was added for 60 min at 37°C. After washing, peroxidase-conjugated anti-mouse IgG (diluted 1 : 1000 in the blocking buffer) was incubated for 60 min at 37°C. After three washings, binding of C1q to anti-TPO aAb was detected by addition of 4 mg ml^−1^ 2-phenylenediamine solution containing 0.03% (v/v) hydrogen peroxide in 0.1 M citrate buffer (pH 5.0). The reaction was stopped with 2 M H_2_SO_4_ and the resulting absorbance measured at 490 nm.

### *In vitro* complement-dependent cytotoxicity assay

To test complement-mediated cytotoxicity, NPA cells (10^6^) were labelled with 100 *μ*Ci ^51^Cr for 60 min at 37°C. After washing, labelled target cells were distributed (2 × 10^4^ cells per well) in 96-well U-bottomed culture plates. Target cells (triplicate wells) were incubated (4°C, 45 min) in culture medium in the absence or presence of an anti-TPO aAb (50 *μ*g ml^−1^) (recombinant B4, B4′, aAbs purified from sera of patients suffering from AITD or monoclonal antibodies (mAb15 and mAb59) known to target two different regions on TPO surface (Ruf *et al*, 1989)). Cells were incubated for 4 h at 37°C, with a source of Complement (guinea pig serum; Sigma-Aldrich). After centrifugation (5 min, 1000 g), aliquots (100 *μ*l) of the supernatant were sampled for *γ*-counting. Cytotoxicity was expressed and calculated as previously described.

### Papillary thyroid cancer cell proliferation assay

NPA cell growth was assessed after 5 days of culture using the cell bromodeoxyuridine (BrdU) proliferation kit (Roche) according to the manufacturer's instructions. NPA cells were cultured in triplicate in 96-well culture microplates at 2 × 10^4^ cells per well (Techno plastic products, Trasadingen, Switzerland) in 200 *μ*l of culture medium in the absence or presence of 50 *μ*g ml^−1^ of anti-TPO recombinant aAbs (B4 or B4′) or anti-TPO aAbs from patients' sera used as inhibitors. Microplates were then incubated for 4 days at 37°C in a wet atmosphere containing 5% CO_2_. Bromodeoxyuridine 1 : 100 dilution was then added, and microplates incubated for an additional 24 h. Bromodeoxyuridine incorporation was measured using a horseradish peroxidase-conjugated anti-BrdU antibody and *o*-phenylenediamine (100 *μ*l per well) as substrate. The colorimetric reaction was stopped by addition of 50 *μ*l of 4 N sulphuric acid per well and absorbance measured at 490 nm.

## Results

### Thyroid cancer and effector cell lines characterisation

**Figure 1:**
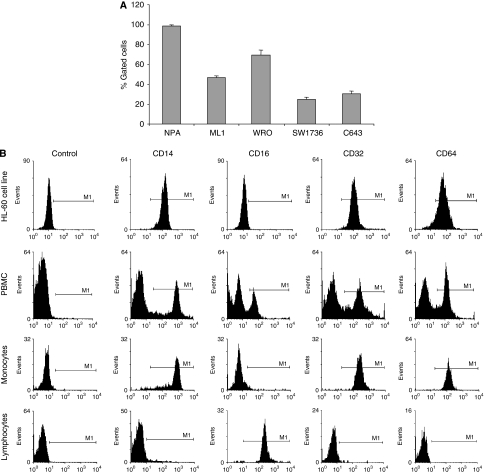
Thyroid cancer and effector cell lines characterisation. (**A**) TPO expression was investigated by flow cytometry on papillary (NPA), follicular (ML1 and WRO) and anaplastic thyroid cancer (SW1736 and C643) cell lines. Cells were incubated successively with a human anti-TPO antibody and with an anti-human FITC antibody. Data correspond to the average of two experiments. (**B**) Expression of four specific markers (CD14, CD16, CD32 and CD64) was studied on effector cells: peripheral blood mononuclear cells (entire PBMC, lymphocytes and monocytes) and a monocyte cell line (HL-60). CD14 is only expressed on monocytes; Fc*γ* receptors CD16 on lymphocytes, CD32 and CD64 on monocytes. Conjugated controls show no background staining.

Before testing the potential use of anti-TPO aAbs for immunotherapy of thyroid cancer by cytotoxic assays (ADCC and CDC), we first characterised the target and effector cells. Expression of thyroperoxidase was investigated in five potential target cells, human anaplastic (SW1736 and C643), follicular (ML1 and WRO) and papillary (NPA) thyroid cancer cell lines. Thyroperoxidase was found to be expressed in 100% of papillary thyroid cells line (NPA) *vs* in only 43 and 69% of, respectively, ML1 and WRO follicular thyroid cancer cells. As expected human anaplastic cancer cells (SW1736 and C643) poorly expressed TPO on their cell surface (Figure 1A). Various populations of effector cells exert different functions by FcR-mediated antibody-antigen binding. Fc*γ*RI (CD64) (the high-affinity IgG receptor), Fc*γ*RIIa (CD32) and Fc*γ*RIII (CD16) (the low-affinity IgG receptor) are activating receptors present on a wide range of myeloid cells (monocytes, macrophages, dendritic cells, neutrophils and natural killer (NK) cells). As we previously showed (Rebuffat *et al*, 2008) that monocytes are potential effector cells in AITD, the monocyte cell line HL-60 and PBMC were chosen as effector cells for cytotoxic assays. We characterised these cells regarding monocytes surface markers such as CD14 and Fc*γ*R expression. The differentiation of HL-60 into monocytes with D_3_ vitamin was assessed by determining CD14 expression (98.26%). Flow cytometry analysis showed that Fc*γ*RII (CD32) and Fc*γ*RI (CD64) are expressed on the HL-60 cell line and on the monocyte population present in PBMC (more than 90% of cells were stained). On the other hand, Fc*γ*RIII (CD16) was only present on the lymphocyte population of PBMC and more specifically on NK cells (Figure 1B).

### Human recombinant anti-TPO aAbs B4, B4′ and patients' sera exhibit different binding properties to Fc*γ*Rs and ADCC activity

**Figure 2:**
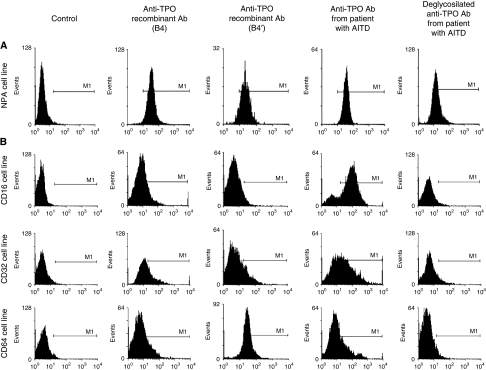
Binding capacity of human recombinant anti-TPO aAbs. (**A**) Three different human recombinant anti-TPO aAbs (anti-TPO aAbs expressed in baculovirus (B4), in CHO (B4′) and patients' sera) were analysed by flow cytometry for their ability to bind to the NPA cell line. (**B**) Binding of TPO/anti-TPO complexes on Fc*γ*RI (CD64), Fc*γ*RII (CD32) and Fc*γ*RIII (CD16) expressed by CD cell lines has been analysed by flow cytometry.

**Figure 3:**
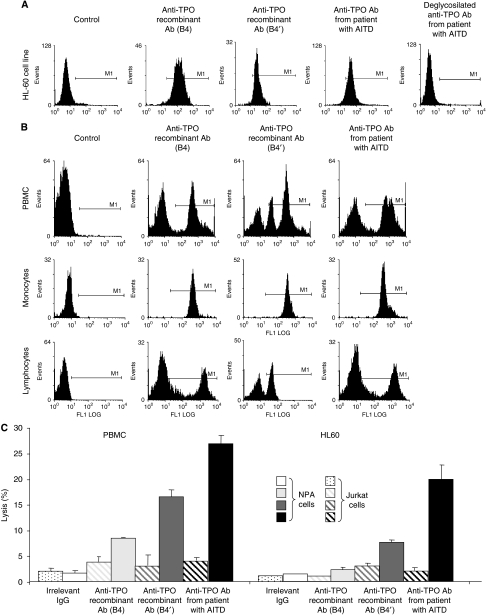
Antibodies-dependent cellular cytotoxicity (ADCC) mediated by human recombinant anti-TPO aAbs (B4 and B4′) and patient's sera anti-TPO aAbs. (**A** and **B**) Binding of TPO/anti-TPO complexes on effector cells (HL-60 cell line and PBMC) was analysed by flow cytometry. (**C**) Antibodies-dependent cellular cytotoxicity was determined by the ^51^Cr release assay. Human thyroid cells were incubated with irrelevant Abs (□) or human anti-TPO aAbs purified from sera of patients suffering from AITD (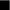), B4 (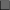) and B4′ (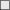) with different effector cells (PBMC and HL-60). *Y* axis presents the percentage of Jurkat (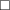) or NPA cells (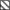) lysis. Bars, s.d. Results are mean of triplicates.

Before studying the ability of human recombinant anti-TPO aAbs B4 and B4′ and affinity purified IgG from patients' sera to mediate ADCC, we evaluated their binding capacity. Therefore, we measured binding of aAbs to TPO expressed on NPA cells (Figure 2A), and binding of TPO/anti-TPO complexes on Fc*γ*RI (CD64), Fc*γ*RII (CD32) and Fc*γ*RIII (CD16) expressed by both CD cell lines (Figure 2B) and effector cells (Figure 3A and B). As indicated in Figure 2A, similar cytometry patterns, showing the binding of anti-TPO aAbs on NPA target cells, occurred with human recombinant anti-TPO aAbs B4 and B4′ (91.96 and 98.77%, respectively). This shows that the expression system used for the anti-TPO aAb (baculovirus/insect cell or in CHO cell) does not modify specificity of the aAb. Furthermore, anti-TPO aAbs purified from patients' sera, which are polyclonal and recognise different epitopes on the TPO molecule, exhibit a comparable binding profile (99.97% of stained cells). The reactivity of complexes of the human recombinant anti-TPO aAbs and patients' sera with TPO was analysed using cell lines 11A1.6 CD64^+^, CDw32-L CD32^+^ and Jurkat CD16^+^, which express only one type of Fc*γ*R (CD16, CD32 or CD64). Only anti-TPO aAbs from patients' sera were able to bind the three types of Fc*γ*Rs with a staining value of 30.99, 54.21 and 81.69% for CD64, CD32 and CD16, respectively. The two human recombinant anti-TPO aAbs (B4 and B4′) exhibited no major differences in binding profile for CD32 and CD16. However, B4′ exhibited a stronger binding affinity than B4 (83.19 *vs* 22.66%) for CD64 (Figure 2B). An anti-Fc*γ*Rs Ab, used as control, abolished the binding of the immune complex to the effector cells and confirmed that binding of human anti-TPO aAbs involves their Fc region (data not shown). Finally, if Ab deglycosylation only moderately alters antigen binding, it significantly modifies the binding of Fc*γ*R on human cell lines (Figure 2B). Taken together, these data suggest that the TPO-anti-TPO aAbs were able to bind Fc*γ*R of cell lines expressing CD16, CD32 and CD64 (Figure 2B).

The next step was to examine the binding of these complexes on effector cells, lymphocyte and monocyte populations present in PBMC as well as HL-60 cells used in ADCC assays. The anti-TPO aAbs (patients' sera, B4 and B4′) bound HL-60 cells by their Fc domain (Figure 3A). However, binding of the aAbs on PBMC showed different patterns due to the level of Fc*γ*Rs expression on these cell populations. The anti-TPO aAbs purified from patients' sera and B4 expressed in baculovirus/insect cell system, which appeared able to bind the three Fc*γ*R types (Figure 3B), strongly interacted with the monocyte and lymphocyte populations present in PBMC. In contrast, the human recombinant anti-TPO aAbs expressed in CHO cells (B4′) bound 90% of the monocyte population but only 40% of the lymphocyte population (most probably NK cell lymphocytes). Thereafter, the ability of recombinant and circulating anti-TPO aAbs to lyse papillary thyroid cancer cells was explored using systems detecting ADCC with the monocyte cell line HL-60 and PBMC as effector cells and NPA cells as targets, because 100% of these cells express TPO on their cell surface. The capacity of recombinant anti-TPO aAbs (B4 and B4′) and purified aAbs from patients' sera to exhibit ADCC was tested (Figure 3C). Anti-TPO aAbs purified from patients' sera lysed 24–29% of NPA cells, whereas the human recombinant anti-TPO aAbs were less effective (B4 (3.5–10%) and B4′ (9–19.5%)). It is worth noting that Jurkat cells, used as a control, were insensitive to anti-TPO aAbs, showing the specificity of these antibodies to lyse NPA cells. Interestingly, whatever the aAbs used (patients' sera, B4 or B4′), a greater ADCC efficiency was obtained with PBMC than with the monocyte cell (29% for PBMC *vs* 24% for HL-60). This result is in accordance with the expression by PBMC of three types of Fc*γ*Rs (CD16 on lymphocytes NK cells, CD32 and CD64 on monocytes) whereas monocyte cells HL-60 express only Fc*γ*RI (CD64) and Fc*γ*RII (CD32). From the comparison of the ADCC activity of the different anti-TPO aAbs, it appears that the anti-TPO aAbs purified from patients' sera were more efficient than the recombinant aAbs expressed in CHO system (B4′) and finally the anti-TPO aAbs expressed in baculovirus/insect cell system (B4). This shows once again that the carbohydrate residues are crucial for the cytotoxic activity of the Abs.

### Human recombinants anti-TPO aAbs B4, B4′ and patients' sera mediate CDC on NPA cells

**Figure 4:**
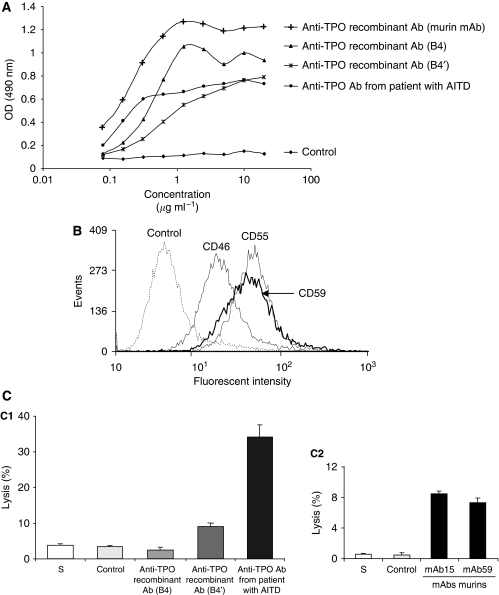
Complement-dependent cytotoxicity mediated by human recombinants (B4 and B4′) and patients' sera anti-TPO aAbs. (**A**) The C1q component of Complement was able to bind to TPO/anti-TPO aAbs complexes but not to an irrelevant IgG. Binding was determined by ELISA. Results are mean of duplicates. (**B**) Expression of complement regulatory proteins CD46, CD55 and CD59 was investigated on NPA cells, by flow cytometry and the data presented in histograms were overlaid. (**C**) Complement-dependant cytotoxicity was determined by the ^51^Cr release assay. *Y* axis presents the percentage of NPA cells lysis. NPA cells were lysed by the complement in presence of human (**C1**) or murin anti-TPO aAbs (**C2**). Bars, s.d. Results are means of duplicates.

Activation of the Complement membrane attack complex (MAC; C5b-9) can lead to direct lysis of target cells by complement-dependent cytotoxicity (CDC). This can occur by the classical pathway with the C1 component, after binding of antibodies to the target cells. To determine whether human recombinants and patients' sera anti-TPO aAbs act through a CDC mechanism on NPA cells, we investigated if C1q component binds to the immune complex anti-TPO aAbs/TPO. As expected, our ELISA data show that C1q does bind to the different complexes (Figure 4A). In contrast, an irrelevant IgG used as control failed to bind the C1q. As tumour cells often evade complement activation because they express membrane-bound complement regulatory proteins, we investigated the relative surface expression of three of the main complement regulatory proteins: CD46 (membrane cofactor protein), CD55 (decay-accelerating factor) and CD59 (protecting) by flow cytometry on NPA cells (Figure 4B). NPA cells expressed all the three proteins, with a greater expression of CD59 and CD55 than CD46 proteins, suggesting that NPA tumour cells were obviously protected against CDC. The ability of Complement to destroy papillary tumour cells was investigated. About 34% of cell lysis (Figure 4C1) was observed when ^51^Cr-labelled NPA cells were incubated with human anti-TPO aAbs purified from patients' sera and guinea pig serum. A lower CDC was observed with recombinant anti-TPO aAbs B4′ (9% of cell lysis), whereas no complement cytotoxic activity was detected using recombinant anti-TPO aAbs B4. Finally, with regard to the CDC activity for the three human (recombinants and patients' sera) anti-TPO aAbs, we used two mAbs (mAb15 and mAb59) directed against two distinct epitopes on the TPO molecule to determine if the epitope recognition by anti-TPO Abs affects CDC mechanism. Figure 4C2 shows a weak and similar CDC with both mAb15 and mAb59 (7 and 9% cell lysis, respectively) showing that, in our case, the epitopic recognition has no function in anti-TPO Abs CDC activity; thus differences observed between anti-TPO aAbs (B4, B4′ and patients' sera) could be due to the polyclonality of the latter.

### Anti-TPO aAbs partially inhibit NPA cells proliferation

**Figure 5:**
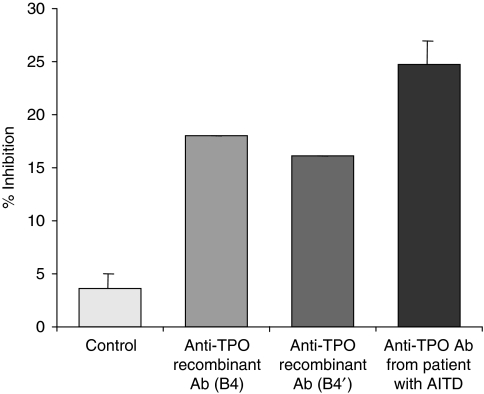
Anti-proliferative effects of anti-TPO aAbs on NPA cells. Analysis of growth inhibition of NPA cells cultured for 5 days with recombinants (B4 or B4′) or patients' sera anti-TPO aAbs as inhibitors. Growth was assessed by incorporation of BrdU. Results are representative of three independent experiments.

The growth of NPA cells cultured in the presence of the anti-TPO recombinant aAbs (B4 and B4′) and anti-TPO aAbs from sera of patients suffering from AITD was partially inhibited (18, 16 and 25%, respectively) but not in control cells cultured in medium alone (Figure 5).

## Discussion

We previously showed that TPO aAbs are, through both ADCC and CDC, able to damage human thyroid cells on binding to TPO expressed on the cell surface (Rebuffat *et al*, 2008). In this study, we hypothesised that human recombinant anti-TPO aAbs could be used to destroy thyroid tumour cells and thus to develop a complementary therapeutic approach in thyroid cancers. Before testing anti-TPO Abs in *in vivo* trials, we compared the *in vitro* cytotoxic activities of baculovirus-expressed, CHO-expressed human IgG1 anti-TPO aAbs named B4 and B4′ with those of purified anti-TPO IgG of patients' sera, on papillary thyroid cancer cells expressing TPO. In this study, we show that anti-TPO aAbs, purified from patients' sera and CHO-expressing human recombinant B4′ aAbs are able to induce moderate CDC, ADCC as well as anti-proliferative effects on NPA cells. In contrast baculovirus-expressing human recombinant B4 displayed no or only minor cytotoxic activities.

We focused this study, until now the only one, on the possible use of anti-TPO aAbs in thyroid cancer immunotherapy to improve the efficiency of conventional treatments and especially in carcinoma that do not respond to radioiodine therapy. In this respect the human anti-TPO aAbs (patients' sera and B4′ aAbs expressed in CHO) tested here exhibit some cytotoxic properties. Their specificity for TPO in targeting thyroid cancerous cell, their capacity to bind the C1q complement and their simultaneous recruitment of immune effector cells by binding to Fc*γ*R through their Fc region agree with the concept that they could be considered as potential tools for passive immunotherapy.

If purified anti-TPO aAbs present in patients' sera, and to a lesser extend B4′ (anti-TPO aAbs expressed in CHO), mediate cytotoxic activity against thyroid cells, only partial or no effects were triggered by B4 (anti-TPO aAbs expressed in baculovirus). The low ADCC activities are probably due to the low glycosylation level of aAbs expressed in baculovirus. Indeed, the presence of specific oligosaccharide structures linked to the C*γ*2 domain of the Fc fragment has been reported to affect the biological activity of the antibody (Jefferis *et al*, 1998; Lifely *et al*, 1995; Wright and Morrison, 1997) by influencing the interaction with Fc*γ*Rs (Tao and Morrison, 1989). In this context, biophysical and molecular studies have shown that N-acetylglucosamine (GlcNac) in the triantennary N-glycan seems also required to induce ADCC. The baculovirus-expressing system used does not incorporate GlcNac in proteins, which probably accounts for the lower cytotoxic activity mediated by the baculovirus-expressed anti-TPO aAb B4.

The biological responses triggered on Fc*γ*Rs stimulation depend on the nature of cells expressing the receptor rather than on the receptor itself. Antibody-dependent cell-mediated cytotoxicity functions have been shown to be shared by various cell populations, including monocytes, NK cells and granulocytes (Ortaldo *et al*, 1987; Steplewski, 1993). Here, we used PBMC. A significant and more efficient ADCC has been obtained using human anti-TPO aAbs from patients' sera and PBMC. This result is probably due to (1) the use of correctly glycosylated Abs (as discussed above) and (2) the expression of all three types of Fc*γ*Rs: Fc*γ*RI (CD64), Fc*γ*RII (CD32) and Fc*γ*RIII (CD16) on the different cell populations in PBMC. Indeed, HL-60 cells express only Fc*γ*RI and Fc*γ*RII, whereas PBMC include lymphocytes NK cells (bearing the Fc*γ*RIII), monocytes and granulocytes (bearing Fc*γ*RI and Fc*γ*RII). These data show that anti-TPO aAbs are able to mediate higher ADCC with PBMC as effector cells.

Complement-dependent cytotoxicity activity depends on the binding of C1q on immune complexes and can be modulated by the presence on tumour cell of membrane-bound complement-regulated proteins. All the anti-TPO aAbs we used bind to C1q but differences are observed in their CDC activity; a maximal efficiency is obtained using anti-TPO aAbs purified from patients' sera. One possible explanation is the polyclonality of the anti-TPO aAbs purified from patients' sera resulting in (1) recognition of a larger number of epitopes of TPO on target cells and (2) deposition of an increased number of complement fragments on these tumour cells, after activation of the complement system. Furthermore, in classical complement activation pathway, CD55 (Bjorge *et al*, 1997) and CD59 (Brasoveanu *et al*, 1996; Gorter *et al*, 1996) have the most prominent function in regulating the MAC, thus preventing cell lysis. These regulatory proteins are expressed on NPA cell membrane and probably partially protect them from CDC. Combining the various human recombinants anti-TPO aAbs we selected by phage display and characterised in term of epitopes (Bresson *et al*, 2003, 2004; Chapal *et al*, 2000; Rebuffat *et al*, 2006), could make it possible to improve complement activation.

Finally, the use of a mix of anti-TPO aAbs resulting in the simultaneous presence of multiple non-competing TPO epitopes should also improve detection of TPO on cancer cell surface, an essential feature for thyroid cancer treatment. It is now well established that the density of target antigen expressed at the cell surface is essential to obtain a pronounced cytotoxic activity. If compared with the HER-2 antigen in breast cancer, TPO is less expressed in NPA cells, explaining the low levels of complement-, cell-mediated cytotoxicity and anti-proliferative effects we observed. However, studies concerning TPO expression in thyroid cancer yielded discrepant data; some studies reported an inverse correlation between TPO and proliferative cell membrane antigen (Garcia *et al*, 1998), whereas others pointed to a normal TPO expression in the majority of thyroid carcinomas (more than 65%) (Czarnocka *et al*, 2001). Using flow cell sorting technique, we analysed TPO expression on papillary (NPA), follicular (ML1 and WRO) and anaplastic (SW1736 and C643) human thyroid cancer cell lines, and showed that TPO is present on all the cell lines tested even though it was found less expressed in anaplastic and follicular thyroid cancer when compared to papillary carcinoma. These results agree with the study of Czarnocka *et al* (2001) showing that TPO is still expressed on thyroid cancer cells but not with the study of Garcia *et al* (1998). These conflicting data could result from differences in the methods and anti-TPO Abs used to detect TPO. Indeed, Garcia *et al* (1998) investigated TPO expression in a series of thyroid tumours by immunostaining using the anti-TPO mAb47 (Ruf *et al*, 1989). We have previously shown that lysine 713 has a critical function for TPO epitope recognition by mAb47 (Bresson *et al*, 2004), which is probably strongly impaired by cross-linking of the amino acid on formaldehyde slide fixation. Czarnocka *et al* (2001) used a TPO capture method that has the advantage to preserve integrity of the antigen structure and thereby allows immunological detection.

Currently, numerous efforts are being made to develop immunological tools for immunotherapy. The presence of TPO in various thyroid carcinoma and metastases, but not in the other tissues, makes it tempting to target thyroid cancer cells with specific anti-TPO Abs.

Our *in vitro* data show that anti-TPO aAbs do exhibit some capacities to destroy NPA thyroid tumour cells by ADCC or CDC but in the present state, cannot be considered as suitable for thyroid cancer immunotherapy. Progress has to be made in improving anti-tumour capacities of these anti-TPO recombinant aAbs. This is the matter of our present investigations, with the engineering of CD16/anti-TPO bi-specific aAbs able to physically cross-link immune and tumour cells and thereby to improve cytotoxic activity.
